# GGDA-net: geometry-guided deformable attention network for Alzheimer’s disease image classification

**DOI:** 10.3389/fnins.2026.1838681

**Published:** 2026-05-26

**Authors:** Dongyan Zhang, Jincan Zhang, Wenna Chen, Ganqin Du

**Affiliations:** 1 College of Information Engineering, Henan University of Science and Technology, Luoyang, China; 2 The First Affiliated Hospital and College of Clinical Medicine of Henan University of Science and Technology, Luoyang, China

**Keywords:** Alzheimer’s disease, geometry-aware attention, GGDA-net, lightweight neural network, linear deformable convolution

## Abstract

**Background:**

Convolutional neural networks (CNNs) have achieved remarkable success in medical image analysis, including Alzheimer’s disease (AD) classification. However, conventional convolution operations rely on fixed sampling patterns, and most existing attention mechanisms primarily focus on feature responses while neglecting spatial sampling geometry, limiting their ability to capture structural variations in brain images.

**Methods:**

To address these limitations, this paper proposes a Geometry-Guided Deformable Attention Network (GGDA-Net) for medical image classification. The proposed framework integrates Linear Deformable Convolution (LDConv) with a Geometry-Aware (GA) Attention mechanism to jointly model feature semantics and spatial geometry. Specifically, LDConv introduces adaptive spatial sampling through learnable offsets, enabling flexible modeling of geometric deformations in brain structures, while the GA attention exploits the resulting geometric cues to guide the network toward more informative anatomical regions.

**Results:**

The experimental results show that the accuracy rates on the two datasets reached 99.38 and 99.16% respectively, which are superior to the existing most advanced algorithms. At the same time, the model maintains a compact size and has a relatively low computational complexity. These results highlight the effectiveness of feature learning based on geometric perception in medical image analysis and Alzheimer’s disease diagnosis.

## 1 Introduction

AD is the most common cause of dementia in the world and is a progressive irreversible neurodegenerative disease (Targa Dias Anastacio et al., 2022). The prevalence of all-cause dementia is projected to increase from 50 million people in 2010 to 113 million globally by 2050 (Knopman et al., 2021). Magnetic Resonance Imaging (MRI) (Leocadi et al., 2023) is widely used for the clinical assessment of AD, where characteristic structural changes such as progressive brain atrophy can be observed across cortical regions (Pini et al., 2016; Rao et al., 2023). In recent years, machine learning (Kavitha et al., 2022) and deep learning (Ebrahimighahnavieh et al., 2020) methods have significantly improved AD diagnosis by learning discriminative patterns from medical imaging data.

Convolutional neural networks (CNNs) have been widely adopted in medical image analysis, particularly for brain MRI–based Alzheimer’s disease diagnosis. Representative architectures such as AlexNet (Krizhevsky et al., 2017), VGG16 (Simonyan and Zisserman, 2015), ResNet50 (Helaly et al., 2022), and DenseNet (Huang et al., 2017), have achieved excellent performance in several visual tasks such as image classification, target detection (Zhang et al., 2022), and semantic segmentation (Neupane et al., 2021), and have driven significant advances in related technologies. However, despite their widespread success, CNNs still have limitations, most notably their constraints on the shape of the convolutional kernel and the sampling method.

To further enhance feature representation, attention mechanisms have been widely incorporated into convolutional networks. Studies such as Squeeze-and-Excitation (SENetV2) (Narayanan, 2023) focus on channel-wise feature recalibration, while the Bottleneck Attention Module (BAM) (Park et al., 2018) jointly models spatial and channel dependencies. Lightweight attention mechanisms such as ECA-Net (Wang et al., 2020) improve efficiency by avoiding dimensionality reduction, and recent efficient or linear attention methods (Zhuoran et al., 2021) aim to reduce computational complexity and improve scalability. However, most existing attention mechanisms rely solely on feature responses and lack explicit modeling of spatial sampling geometry.

To address these challenges, this paper integrates LDConv-based (Zhang et al., 2024) adaptive sampling with a GA mechanism to jointly model feature semantics and structural information, thereby enhancing representation capability in complex anatomical structures. The main contributions of this study are summarized as follows:

- Linear deformable convolution: Unlike standard convolution, LDConv introduces learnable spatial offsets, enabling the network to adaptively sample and model irregular anatomical structures while maintaining linear parameter growth.
- Geometry-aware attention: GA is proposed to model the interaction between feature responses and spatial sampling cues. By exploiting the geometric offsets generated during deformable sampling, the module guides the network to focus on more informative structural regions.
- Efficient integration: We develop a GGDA-Net by integrating LDConv with the proposed GA module. LDConv enables adaptive spatial sampling through learnable offsets, while GA attention exploits the resulting geometric cues to perform geometry-aware feature reweighting.
- Extensive experimental validation: The proposed method is evaluated on the ADNI dataset for Alzheimer’s disease classification. Experimental results demonstrate improved performance over representative deep learning models. These results validate that incorporating geometry-aware adaptive sampling is beneficial for capturing subtle structural variations in brain images.

The remainder of this paper is organized as follows. Section 2 reviews the relevant work on classifying Alzheimer’s disease using deep learning, with a focus on the shortcomings of convolutional neural networks (CNNs), architectures based on transformers, and attention mechanisms. Section 3 presents the proposed GGDA-Net methodology, including the LDConv module and GA attention mechanism. Section 4 describes the experimental setup, including datasets, implementation details, and evaluation metrics. Section 5 reports and discusses the experimental results, including comparisons with state-of-the-art methods and ablation studies. Finally, Section 6 concludes the paper and outlines future work.

## 2 Related work

Machine learning techniques have been widely explored for Alzheimer’s disease (AD) diagnosis using neuroimaging data. For instance, Ahmed et al. (2022) combined Boruta feature selection with gradient boosting trees (GBT) to achieve high-precision early prediction of AD, although the method still suffers from relatively high computational complexity. Other studies have explored various machine learning techniques, including support vector machines (Sharma et al., 2021), random forests (Aria et al., 2021), and K-means clustering (Ikotun et al., 2023), to analyze neuroimaging features. However, a comprehensive review (Mirzaei and Adeli, 2022) suggests that no single traditional machine learning approach consistently outperforms others across different datasets and tasks. Moreover, these methods generally rely on handcrafted feature extraction, which limits their ability to capture complex patterns in high-dimensional medical images.

With the rapid development of deep learning (DL), convolutional neural networks (CNNs) have become a dominant approach for medical image analysis (Goenka and Tiwari, 2021). Several studies have proposed CNN-based frameworks to improve diagnostic accuracy. For example, AbdulAzeem et al. (2021) introduced an end-to-end CNN framework for AD classification, while Helaly et al. (2022) designed a lightweight CNN architecture to reduce computational complexity and mitigate overfitting. El-Assy et al. (2024) proposed a dual-CNN structure to enhance the classification of AD subtypes and stages, and Hemanth et al. (2019) explored an efficient weight assignment strategy for fully connected layers to reduce computational cost. Neetha et al., based on the DEMNET framework, successively proposed the Borderline-DEMNET (Umalakshmi et al., 2024) and BD^2^EMNET (Neetha et al., 2024) models. Both of these models significantly improved the accuracy of the four-class and five-class classification tasks for Alzheimer’s disease by introducing the Borderline-SMOTE class balancing technique. Özkaraca et al. (2023) further designed a hierarchically dense CNN structure to accelerate model processing. Ahila et al. (2022) proposed a novel enhanced CAD system based on CNN to distinguish normal controls from AD patients. In addition to CNN-based approaches, transformer-based architectures have also attracted increasing attention in medical image analysis due to their capability to model long-range dependencies. For instance, Mahim et al. (2024) proposed a hybrid ViT–GRU model combined with explainable AI techniques, while Hu et al. (2023) developed the VGG-TSwinformer model for short-term longitudinal MCI analysis. These deep learning approaches have significantly improved feature learning capability and classification performance in AD-related image analysis.

Despite these advances, most existing models rely on fixed convolutional sampling patterns, which limit their ability to capture geometric deformations in complex brain structures. Furthermore, brain MRI images often exhibit intricate anatomical structures, irregular spatial patterns. Existing attention mechanisms primarily focus on feature responses while neglecting spatial sampling geometry, thereby restricting their capacity to model structural information in medical images. To address these limitations, this paper proposes a lightweight classification framework. Specifically, LDConv enables adaptive spatial sampling through learnable offsets, effectively capturing structural variations across different classes, while the GA attention mechanism leverages geometric offsets to guide the network toward discriminative regions.

## 3 Methodology

In this section, the proposed model is explained in detail and then the basic components used are described.

### 3.1 The proposed model

**Figure 1:**
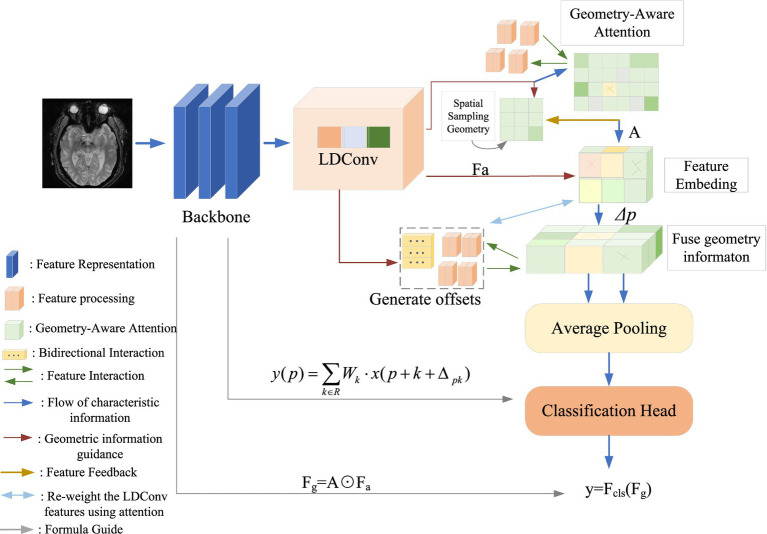
The overall architecture of the proposed GGDA-Net.

The overall framework of the proposed model follows a sequential pipeline that integrates backbone feature extraction, adaptive kernel convolution, geometry-aware attention, and classification, as shown Figure 1. Given an input medical image, the backbone network first extracts hierarchical feature representations, which encode both low-level texture and high-level semantic information. These features are then fed into the LDConv module, which serves as the core component for adaptive spatial modeling. Unlike conventional convolution with fixed sampling locations, LDConv introduces learnable offsets to dynamically adjust the sampling positions, enabling the convolution operation to better align with irregular anatomical structures. Specifically, the convolution process is formulated as *y* (*p*) = ∑*W_k_*·*x* (*p* + *k* + Δ*p_k_*), where Δp_k_ denotes the learnable offset for the k-th sampling point. Within this process, LDConv generates two key outputs: the adaptive feature representation denoted as Fa, and the corresponding spatial offsets that describe the sampling geometry. The feature Fa represents the aggregated feature embedding after adaptive sampling, capturing spatially aligned structural information. Meanwhile, the offset term Δp_k_ encodes the geometric deformation of sampling positions, which is further utilized to model spatial relationships. To enhance the representation capability, The GA Attention module takes both the adaptive feature Fa and the learned offsets Δp as inputs. Specifically, the offsets are further decomposed to extract deformation magnitude, enabling the attention mechanism to capture both spatial geometry and deformation intensity. The attention mechanism produces a geometry-aware weighting map A, which highlights informative regions while suppressing irrelevant responses. This process effectively integrates feature representation and geometric information into a unified representation. Subsequently, a global average pooling layer is applied to aggregate spatial information, followed by a classification head to generate the final prediction y. Through this pipeline, the proposed method achieves the research objective of improving spatial feature representation by combining adaptive sampling and geometry-aware attention, thereby enhancing the model’s ability to capture complex structural patterns in medical images.

Given an input image $X\in R^{C\times H\times W}$ , the backbone network first extracts feature representations. The extracted features are then refined by LDConv to enable adaptive spatial sampling. Based on the learned sampling geometry, GA attention module performs structure-conditioned feature reweighting. Finally, the enhanced features are fed into a classifier for prediction. The entire process can be represented by Equation 1:

$$y=F_{\mathrm{cls}}(F_{\text{GGAD}}(F_{\text{LDConv}}(F_{\text{backbone}}(X))))$$ (1)

### 3.2 Backbone feature extraction

The backbone serves as the primary feature extraction network, responsible for transforming the input image into discriminative feature representations. It extracts hierarchical spatial and semantic information that provides the foundation for the subsequent adaptive convolution and attention modules. Let the input image be denoted as $I\in R^{C_{0}\times H_{0}\times W_{0}}$ , where $C_{0}$ , $H_{0}$ and $W_{0}$ denote the number of channels, height, and width of the input image, respectively.

Through a sequence of convolutional operations, the backbone network aggregates local spatial information and generates a deep feature representation. The convolution operation at spatial location p can be expressed as Equation 2:

$$X(p)=\sum_{n=1}^{N}W_{n}\cdot I(p+p_{n})$$ (2)

where p denotes the spatial position in the feature map, $p_{n}$ represents the predefined sampling offset within the convolution kernel, and $W_{n}$ denotes the corresponding convolution weight.

The resulting feature map $X\in R^{C\times H\times W}$ encodes rich spatial and semantic information, which serves as the input to the proposed LDConv module for further geometry-guided feature learning.

### 3.3 Linear deformable convolution

Compared with standard convolution, LDConv allows convolution kernels with arbitrary numbers of parameters and flexible sampling shapes, providing a richer trade-off between model complexity and representation capability. LDConv modifies the parameter growth trend from quadratic to linear with respect to kernel size. When the number of parameters is set to $K_{2}$ , LDConv degenerates to the standard deformable convolution.

**Figure 2:**
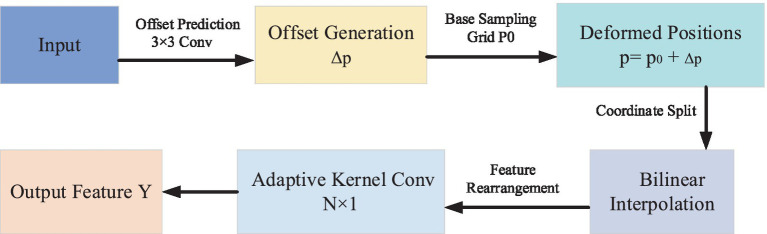
Illustration of the LDConv module with adaptive sampling and feature aggregation.

As illustrated in Figure 2, the LDConv module first predicts sampling offsets using a 3 × 3 convolution layer, generating the offset vector Δp. The offsets are then added to a predefined base sampling grid $p_{0}$ to obtain the deformed sampling positions. Since the resulting sampling coordinates are generally fractional, bilinear interpolation is applied to extract the corresponding feature values from the input feature map. The sampled features are subsequently rearranged to match the convolutional aggregation structure of LDConv. Finally, an ***N × 1*** convolution is applied to aggregate the sampled features and produce the output feature map ***Y***. This mechanism enables adaptive spatial sampling and flexible kernel shapes, allowing the network to better capture geometric variations in complex structures.

Suppose the input feature map is $X\in R^{C\times H\times W}$ , where C denotes the number of channels, and ***H*** and ***W*** are the height and width of the feature map, respectively. For each spatial location p=(h,w) , the convolution operation aggregates information from ***N*** sampling points. Instead of using a fixed ***k × k*** sampling grid, LDConv learns spatial offsets to generate adaptive sampling coordinates. The initial sampling set is defined by Equation 3:

$$P={\left\{p_{n}\right\}}_{n=1}^{N},P_{n}=\{p_{n}^{x},p_{n}^{y}\}$$ (3)

The offset field is predicted from the input feature map, as shown in Equation 4:

$$\mathrm{\Delta p}=f_{\text{offset}}(X),\mathrm{\Delta p}\in {\mathbb{R}}^{2N\times H\times W}$$ (4)

Thus, the adaptive sampling coordinates become (as shown in Equation 5):

$$p_{n}^{\prime }=p+p_{n}+\Delta p_{n}$$ (5)

where, p: center position; p_n_: initial sampling location; Δp_n_: learned offset.

Since the updated coordinates $p_{n}^{\prime }$ may lie on fractional positions, bilinear interpolation is used to sample feature values (as shown in Equation 6):

$$F_{n}(p)=\text{Interpolate}(X,p_{n}^{\prime })$$ (6)

where $\;F_{n}(p)\;$ denotes the sampled feature at the n-th adaptive location.

The sampled features are aggregated using convolution weights (as shown in Equation 7)

$$Y(P)=\sum_{n=1}^{N}W_{n}\cdot F_{n}(p)$$ (7)

where, $W_{n}$ represents the weight of the n-th sampling point.

Through this adaptive sampling mechanism, LDConv dynamically adjusts the spatial receptive field and improves the ability to capture irregular geometric patterns.

### 3.4 Geometry-aware attention

**Figure 3:**
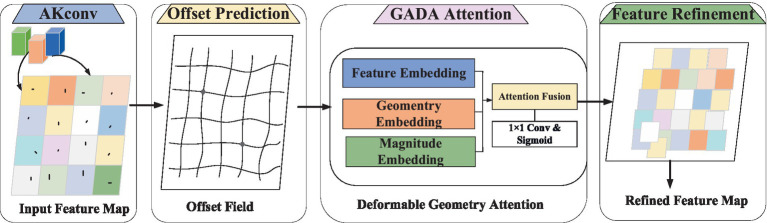
Architecture of the proposed geometry-aware attention (GA) module.

In this paper, the proposed GA module enhances spatial feature representation by explicitly incorporating geometric deformation cues derived from LDConv. As illustrated in Figure 3, unlike conventional attention mechanisms that estimate feature importance solely from feature activations, the GA module jointly models feature responses and spatial sampling geometry. Specifically, the module integrates three complementary components: feature embedding, geometry embedding derived from offset fields, and deformation magnitude embedding, which together capture both semantic information and structural deformation patterns.

The proposed GA module enhances spatial feature representation by explicitly incorporating geometric deformation cues derived from the LDConv module. As shown in Figure 3, unlike conventional attention mechanisms that estimate feature importance solely from feature activations, the GA module jointly models feature responses and spatial sampling geometry. Specifically, the module integrates three complementary components: feature embedding, geometry embedding derived from offset fields, and deformation magnitude embedding, which together capture both semantic information and structural deformation patterns. The feature embedding captures the essential feature characteristics, while the geometry embedding comes from the offsets, which guide the sampling process to adapt to spatial variations. The deformation magnitude embedding represents the strength of deformation, which is crucial for capturing varying patterns of structural transformation.

The embedded representations are fused through a lightweight attention generation unit composed of a 1 × 1 convolution followed by a sigmoid activation to produce geometry-guided attention weights. By explicitly leveraging the geometric offsets generated during deformable sampling, the GA module enables the network to focus on structurally informative regions and better capture irregular spatial patterns. This design introduces structural inductive bias into the attention mechanism, allowing the model to more effectively handle anatomical variability and complex deformation patterns compared with conventional channel or spatial attention mechanisms.

The GA attention is introduced following the LDConv layer. Given the output feature map ***Y*** and the sampling offsets *Δ* produced by LDConv, the attention weights ***A*** are computed using Equation 8:

$$A(p)=\sigma (\phi_{f}(Y)+\phi_{g}(\Delta )+\phi_{m}(M))$$ (8)

where $\emptyset_{f}(.)$ , $\emptyset_{g}(.)$ , $\emptyset_{\mathrm{fm}}(.)$ are lightweight transformations for feature, geometry, and deformation magnitude, respectively, and σ(.) is the sigmoid function.

The refined feature map is then calculated by Equation (9) as follows:

Z=Y⊙A (9)

## 4 Experiment analysis

### 4.1 Data description

All data used in this study were obtained from the Alzheimer’s Disease Neuroimaging Initiative (ADNI) database. Two independent datasets were constructed from different ADNI phases to evaluate the generalization ability of the proposed method. The demographic information is summarized in Table 1.

**Table 1:** Demographic details of different diagnostic groups in ADNI datasets.

ADNI					ADNI2			
Class	Subject	Trian images	Test images	Images	Subject	Trian images	Test images	Images
AD	89	1747	437	2,184	87	1,184	297	1,481
CN	116	2,426	607	3,033	87	1,388	347	1735
MCI	166	3,612	903	4,515	99	1,601	401	2002
SMC	—	—	—	—	64	1,060	266	1,326
Total	371	7,785	1947	9,732	336	5,233	1,311	6,544

#### 4.1.1 Three-class dataset (ADNI)

The first dataset was derived from the complete one-year 1.5 T MRI data available in the ADNI-1 database. Subjects with complete baseline scans and clearly defined diagnostic labels were selected. The final dataset comprised 371 subjects across three diagnostic categories: Alzheimer’s disease (AD, *n* = 89), cognitively normal (CN, *n* = 116), and mild cognitive impairment (MCI, *n* = 166). The original data were stored in 3D NIfTI format. Each 3D volume was sliced along the coronal plane to generate a series of 2D images. Subsequently, each slice underwent window width and level adjustment to optimize contrast, followed by intensity normalization. Finally, all images were uniformly resized to 256 × 256 pixels and saved as PNG format.

#### 4.1.2 Four-class dataset (ADNI2)

To construct a more challenging task that better aligns with fine-grained clinical diagnostic scenarios, a second dataset was built from the ADNI2 database. This dataset introduces the subjective memory complaint (SMC) category, an early-stage class, in addition to AD, CN, and MCI, thereby forming a four-class classification task (AD, *n* = 87; CN, *n* = 87; MCI, *n* = 99; SMC, *n* = 64). The original DICOM files were linearly normalized to a grayscale range of [0, 255]. Blank slices lacking brain parenchyma were excluded. All images were resized to 256 × 256 pixels.

**Figure 4:**
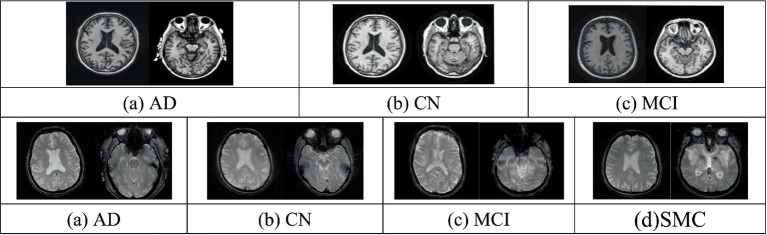
Different types of images on the ADNI datasets.

Both datasets were split at the subject level into training and testing sets with a ratio of 80/20. Notably, as the two datasets originate from different ADNI phases with distinct acquisition protocols and subject cohorts, they naturally constitute a cross-dataset validation scenario. To further mitigate the risk of overfitting and enhance model generalization, data augmentation strategies including random rotation and random horizontal flipping were applied during training. As shown in Figure 4, representative MRI image examples from the ADNI dataset are presented. Top row (ADNI1): (a) AD, (b) CN, (c) MCI. Bottom row (ADNI2): (a) AD, (b) CN, (c) MCI, (d) SMC. All images are provided in PNG format to simplify subsequent processing.

### 4.2 Implementation details

All experiments were conducted on a workstation running Windows 10 with 32 GB RAM and an NVIDIA RTX 4060 Ti GPU (16 GB memory). The proposed model was implemented using the PyTorch deep learning framework. The employed parameters for model training are shown in Table 2.

**Table 2:** Parameters used for model training.

Parameters	Settings
Hardware	GPU, CPU
Framework	PyTorch
Optimizer	Adam
Learning rate	1e-4
Batch size	8
Loss function	CrossEntropyLoss
Epochs	150

During training, the Adam optimizer was employed with an initial learning rate of $1\times 10^{-4}$ . The learning rate was dynamically adjusted using a decay strategy during the training process. The batch size was set to 8, and the model was trained for a maximum of 150 epochs. To improve generalization and prevent overfitting, an early stopping strategy based on validation accuracy was adopted. The cross-entropy loss function was used as the training objective to minimize the classification loss between the predicted outputs and the ground truth labels.

### 4.3 Evaluation metrics

This section firstly focuses on describing the influences on performance in the AD stage that are used to predict the classification of outcomes. The underlying outcomes for assessing the validity of classification systems cover four main categories: true positives (TP), true negatives (TN), false positives (FP), and false negatives (FN). The model performance was evaluated using several key metrics, including Precision (Pre), Recall (Rec), and F1-score (F1). The mathematical expressions for these metrics are shown below in Equations (10)–(13):

$$\text{Accuracy}=\frac{\mathrm{TP}+\mathrm{TN}}{\mathrm{TP}+\mathrm{TN}+\mathrm{FP}+\mathrm{FN}}$$ (10)

$$\text{Precision}=\frac{\mathrm{TP}}{\mathrm{TP}+\mathrm{FP}}$$ (11)

$$\text{Recall}=\frac{\mathrm{TP}}{\mathrm{TP}+\mathrm{FN}}$$ (12)

$$F1-\text{score}=\frac{2\mathrm{TP}}{2\mathrm{TP}+\mathrm{FP}+\mathrm{FN}}$$ (13)

### 4.4 Comparison with classical networks

We compare the proposed model with several widely used deep learning architectures, including AlexNet, VGG16, ResNet50, DenseNet121, and Swin-Transformer, as shown in the Table 3.

**Table 3:** Performance comparison with different deep learning architectures.

Models	Pre (%)	Rec (%)	F1 (%)	Acc (%)	Para (m)	FLOPs (G)
AlexNet	96.89	97.04	96.96	96.61	14.59	**0.309**
VGG16	98.68	98.66	98.67	98.51	134.27	15.470
ResNet50	99.32	99.21	99.26	99.18	23.51	4.132
DenseNet121	99.21	99.14	99.17	99.08	6.957	2.896
Swintransformer	99.30	99.32	99.31	99.23	27.520	4.371
Ours	**99.45**	**99.45**	**99.45**	**99.38**	**3.697**	0.988

Bold values indicate the best performance among all compared methods.

Traditional networks such as AlexNet and VGG16 achieve relatively lower performance and require significantly larger model parameters. Modern architectures including ResNet50, DenseNet121, and Swin-Transformer achieve competitive results with accuracies exceeding 99%. However, the proposed model achieves the best performance, reaching 99.38% accuracy and 99.45% F1-score, while maintaining significantly lower model complexity (3.697 M parameters and 0.988G FLOPs).

**Figure 5:**
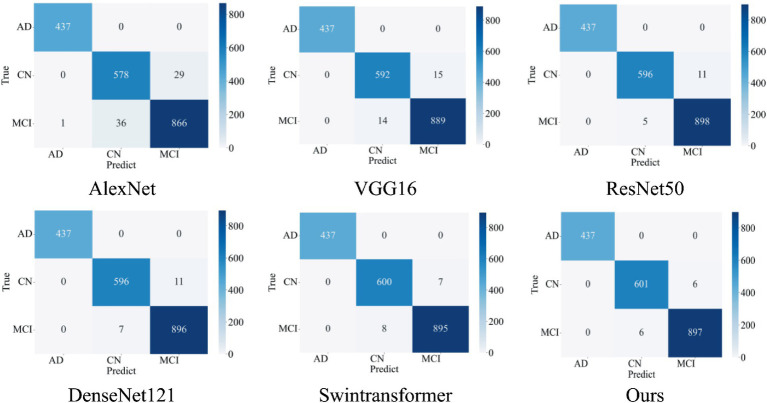
The test confusion matrix of different methods.

Regarding the robustness of the model in the presence of class imbalance, further corroboration is provided by the confusion matrix in Figure 5. The matrix illustrates that a vast majority of instances across every category are correctly classified, with highly concentrated diagonal entries and near-zero off-diagonal misclassifications. This indicates that the model does not exhibit overfitting or bias toward any specific category. Even when a particular class has relatively fewer samples, the model can still accurately distinguish it based on highly discriminative features. Furthermore, from a quantitative perspective, the proposed model achieves perfectly equal precision (99.45%) and recall (99.45%), with the same F1-score of 99.45%. This perfect alignment between precision and recall implies that the model is insensitive to class distribution shifts: the high recall indicates that the model can identify the vast majority of positive samples, while the high precision indicates that the model rarely misclassifies other categories as the target category. Compared to AlexNet (Pre 96.89%/Rec 97.04%) and VGG16 (Pre 98.68%/Rec 98.66%), the proposed model achieves a better balance between precision and recall, further validating its superior robustness under class imbalance conditions.

In summary, the confusion matrix in Figure 5 and the precision-recall consistency collectively demonstrate that the proposed method not only achieves the highest overall classification accuracy but also effectively enhances robustness to class imbalance while maintaining computational economy, thus realizing a favorable trade-off between high accuracy and strong robustness.

### 4.5 Ablation study on convolution architectures

To investigate the impact of different convolution strategies on model performance, several model variants were constructed by replacing the convolution operations while maintaining the same backbone architecture and attention modules (BAM and SENetV2). The evaluated variants include Model-Transpose, Model-Dynamic, Model-Group, and Model-LDConv, and the corresponding results are summarized in Table 4.

**Table 4:** Performance comparison of different convolution architectures.

Models	Convolution	Attention	Pre (%)	Rec (%)	F1 (%)	Acc (%)	Para (m)	FLOPs (G)
Model-Transpose	Transpose Conv	BAM + SENetv2	98.75	98.50	98.62	98.46	4.470	2.249
Model-Dynamic	Dynamic Conv	BAM + SENetv2	98.40	98.23	98.32	98.15	7.960	0.170
Model-Group	Group Conv	BAM + SENetv2	98.93	98.86	98.90	98.77	3.598	0.688
Model-LDconv	LDConv	BAM + SENetv2	99.01	98.92	99.04	98.92	3.733	0.987

Model-Transpose achieves an accuracy of 98.46% but suffers from high computational complexity. Model-Dynamic substantially reduces FLOPs, yet introduces more parameters and yields inferior accuracy. Model-Group offers competitive performance with 98.77% accuracy while maintaining lower computational cost. By incorporating LDConv, which enables adaptive spatial sampling for better modeling of structural variations in medical images, the proposed Model-LDConv achieves the highest accuracy of 98.92% with reduced parameter complexity. Overall, these results highlight the importance of selecting appropriate convolution strategies.

### 4.6 Ablation study on attention modules

To further investigate the role of attention mechanisms in deformable feature modeling, we conduct an ablation study by progressively introducing LDConv (L), BAM (B), and SE (S), and the proposed GGDA-Net. The experimental results are presented in Table 5.

**Table 5:** Ablation results of different attention mechanisms.

Model								
LDConv (L)	BAM (B)	Se (S)	Pre (%)	Re (%)	F1 (%)	Acc (%)	Para (m)	FLOPs (G)
×	×	×	98.29	98.22	98.25	98.05	4.421	2.222
√	×	×	96.52	96.49	96.51	96.10	4.074	1.835
√	√	×	96.46	96.07	96.25	95.84	3.711	0.986
√	×	√	98.66	98.47	98.56	98.41	3.706	0.960
×	√	√	98.34	98.14	98.24	98.05	4.470	2.249
√	√	√	99.01	98.92	99.04	98.92	3.733	0.987

The baseline model achieves 98.05% accuracy. When LDConv is introduced, the performance drops to 96.10%, indicating that although deformable convolution enhances geometric representation ability, the additional spatial flexibility may also introduce unstable feature responses without proper feature calibration.

To stabilize the deformable representations, conventional attention mechanisms are further explored. Incorporating SE attention together with LDConv improves the accuracy to 98.41%, demonstrating that channel-wise recalibration can partially regulate deformable features. In contrast, combining BAM with LDConv does not yield further improvement, suggesting that traditional spatial–channel attention is not fully compatible with geometry-aware representations. Moreover, stacking BAM and SE without LDConv shows limited benefit compared with the baseline, implying that conventional attention mechanisms are not sufficient to capture deformation-related information.

**Figure 6:**
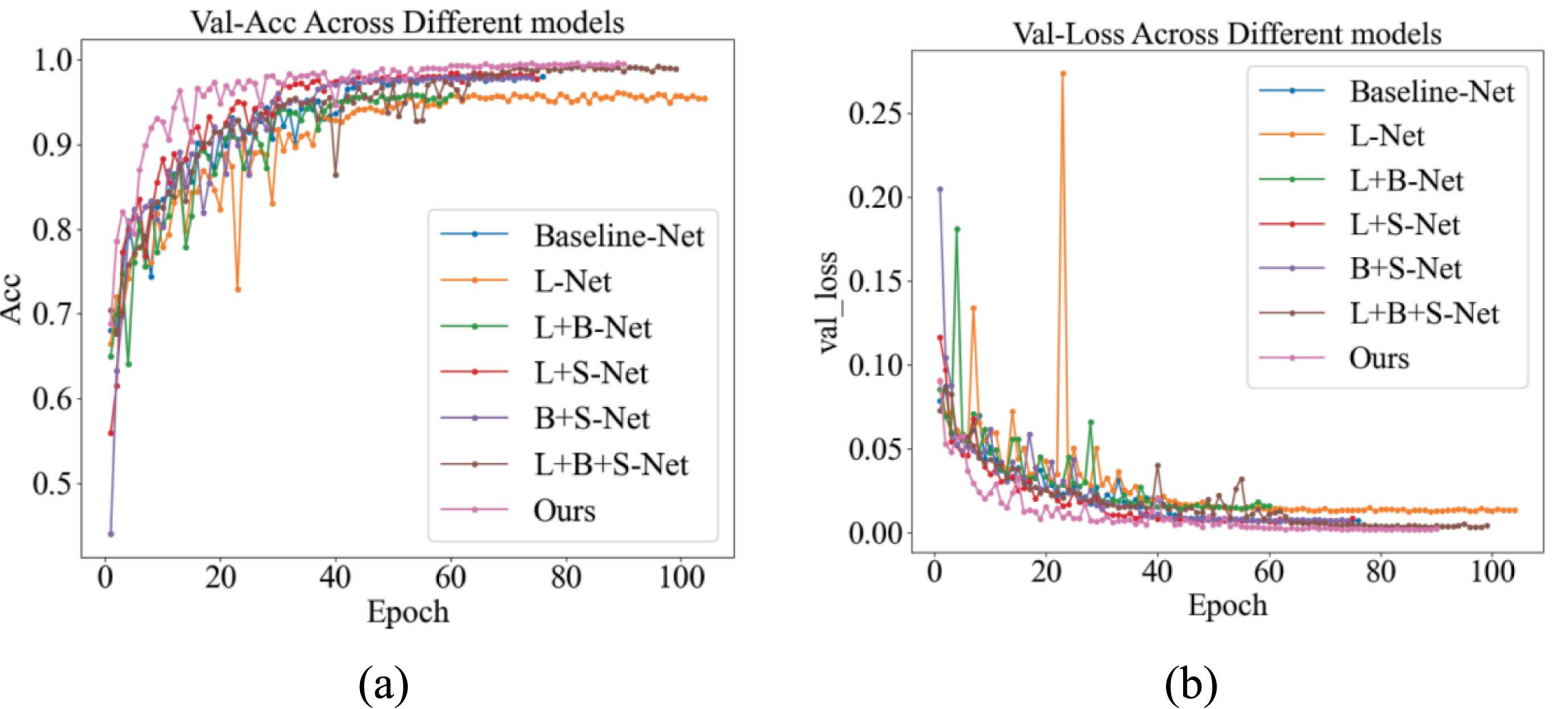
Results description of accuracy-loss curves.

Motivated by these observations, we introduce the Geometry-guided Deformable Attention (GGDA) module, which explicitly incorporates geometric offsets and deformation magnitude into the attention computation. With the proposed design, the final model achieves the best performance with 99.38% accuracy and 99.45% F1-score, demonstrating the effectiveness of geometry-aware attention for deformable feature learning. The results of the ablation study are shown in Figure 6, where (a) compares validation accuracy (Val-Acc) across different models, and (b) compares validation loss (Val-Loss) across different models. These results suggest that attention mechanisms for deformable convolution should explicitly consider geometric deformation information, which motivates the design of the proposed GGDA module.

### 4.7 Ablation study on LDConv and GA attention modules

To further investigate the individual contribution of each component, ablation experiments were conducted on LDConv and GA attention, as shown in Table 6. The baseline model, which employs standard convolution, achieves an accuracy of 98.05%. When LDConv is introduced alone, the accuracy slightly decreases to 96.10%, which can be attributed to the additional difficulty of learning spatial offsets in deformable convolution. When GA attention is incorporated into the baseline model alone, the accuracy improves to 98.92%, demonstrating that GA attention effectively enhances feature representation. Notably, when LDConv and GA attention are jointly applied, the model achieves its best performance with 99.38% accuracy, while simultaneously reducing model complexity to 3.697 M parameters and 0.988G FLOPs.

**Table 6:** Ablation results of different module combinations.

Model	Pre (%)	Re (%)	F1 (%)	Acc (%)	Para (m)	FLOPs (G)
Baseline	98.29	98.22	98.25	98.05	4.42	2.22
LDconv	96.52	96.49	96.51	96.10	4.074	1.835
Baseline+GA	98.98	99.04	99.01	98.92	4.435	2.261
Ours	99.45	99.45	99.45	99.38	3.697	0.988

These results indicate that GA attention effectively exploits the geometric information encoded in the offsets generated by LDConv, guiding the network to focus on more discriminative regions and thereby improving both model performance and efficiency. Specifically, LDConv provides geometric priors through adaptive sampling, while GA attention reweights these geometric cues, forming an effective synergistic mechanism.

### 4.8 Multi-class classification results

**Figure 7:**
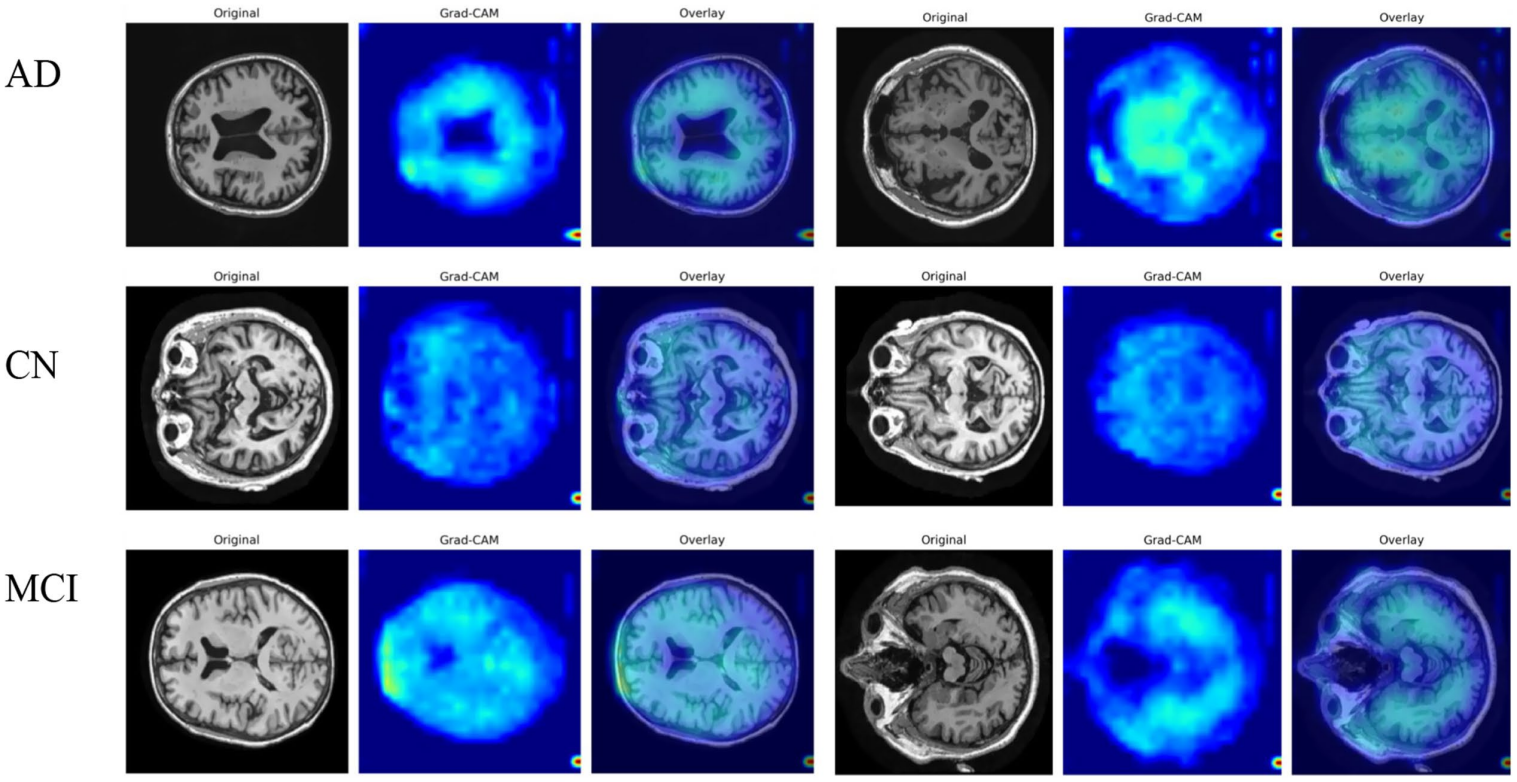
Grad-CAM visualization results of the proposed GGDANet model.

To further evaluate the robustness of the proposed model, experiments were conducted on both three-class and four-class classification tasks. Table 7 presents the performance of the proposed model on three-class and four-class classification tasks. Figure 7 presents the Grad-CAM visualization results for the proposed model. The highlighted regions indicate the areas that contribute most to the model’s prediction.

**Table 7:** Performance comparison of the proposed model on three-class and four-class classification tasks.

Task	Class	Pre (%)	Rec (%)	F1 (%)	Acc (%)
3-class	AD	100.00	100.00	100.00	99.38
	CN	99.01	99.01	99.01	
	MCI	99.34	99.34	99.34	
4-class	AD	99.33	99.33	99.33	99.16
	CN	99.42	99.14	99.28	
	MCI	98.52	99.50	99.01	
	SMC	99.62	98.50	99.05	

For the three-class task, the proposed model achieves excellent performance, with 100% precision, recall, and F1-score for the AD class. The CN and MCI classes also obtain high F1-scores of 99.01 and 99.34%, respectively, leading to an overall accuracy of 99.38%. For the more challenging four-class task, the model still maintains strong performance with 99.16% accuracy, achieving F1-scores of 99.33, 99.28, 99.01, and 99.05% for AD, CN, MCI, and SMC, respectively. The slight performance decrease in the four-class task is expected due to the increased classification difficulty.

It is worth noting that even in the four-class task where class sample sizes may be imbalanced (e.g., the SMC class often has fewer samples), the proposed model maintains highly consistent precision and recall across all classes. For instance, the SMC class achieves a precision of 99.62% and a recall of 98.50%, which are very close to each other. Similarly, the MCI class achieves a precision of 98.52% and a recall of 99.50%, also demonstrating a good balance. This consistency between precision and recall indicates that the model does not bias toward majority classes or neglect minority classes due to potential imbalanced data distributions. In other words, the model exhibits strong robustness to class distribution shifts, enabling fair and accurate discrimination across all classes even when sample sizes vary. These results collectively demonstrate that the proposed model not only excels in the three-class task but also exhibits high robustness and strong discriminative capability in the more complex four-class task. As shown in the Figure 7, the model mainly focuses on meaningful brain regions related to Alzheimer’s disease, demonstrating that the network can capture discriminative pathological features from MRI images. This visualization further confirms the interpretability and reliability of the proposed method.

**Figure 8:**
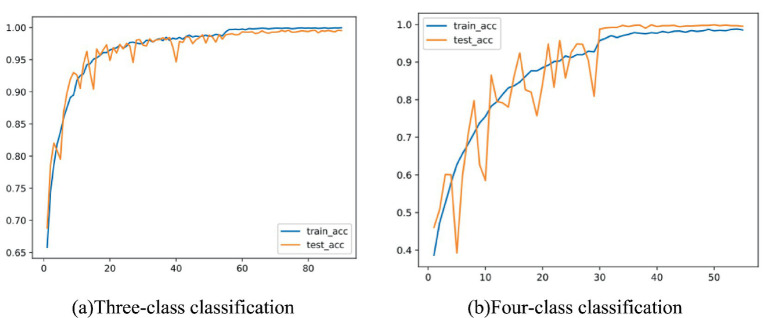
Training and validation accuracy curves for the three-class and four-class tasks.

Figure 8 shows the curves of training accuracy and test accuracy, where (a) presents the three-class classification results, and (b) presents the four-class classification results. In the three-class task, training accuracy increased from approximately 65 to 99%, while validation accuracy rose from 68 to 99%. The two curves remained highly synchronized throughout most epochs, with validation accuracy slightly higher than or comparable to training accuracy. In the four-class task, training accuracy improved from 38 to 99%, and validation accuracy from 46 to 99.2%, with only minor and transient fluctuations in a few epochs. The close alignment of the two curves indicates no significant overfitting, a stable learning process, and strong generalization capability.

## 5 Discussion

Compared with state-of-the-art models in Table 8, the proposed method achieves superior or competitive performance against existing approaches. The proposed GGDA framework improves the capability of the network to model complex structural features.

**Table 8:** Comparative analysis with the state-of-the-art techniques.

Number of classes	References	Methods	Acc (%)	Pre (%)	Rec (%)	F1 (%)
4-class	Loddo et al. (2022)	Deep-Ensemble	97.71	—	—	—
	Al-Adhaileh (2022)	AlexNet model	94	—	—	—
	Papanna Umalakshmi et al. (2024)	ADEMNET architecture	95.45	—	—	—
	Choudhury et al. (2024)	Coupled-GAN	94.5	95	—	94
	Hassan et al. (2025)	SCCAN	99.22	99.21	99.21	99.22
	Yilmaz and Kuş (2026)	GA-optimized	96.43	96.50	96.44	96.45
	Ours	GGDA-Net	**99.28**	99.22	99.11	99.17
3-class	Choi and Lee (2020)	DCNN ensemble	93.84	—	—	—
	Chen et al. (2025)	MMDF	97.65	96.98	96.40	96.69
	Haq et al. (2025)	CNN-LSTM	92.3	92.1	—	92.25
	Ours	GGDA-Net	**99.38**	99.45	99.45	99.45

Bold values indicate the best performance among all compared methods.

For the three-class task, the proposed model achieves 99.38% accuracy, along with 99.45% precision, recall, and F1-score, comprehensively outperforming all compared methods. Specifically, compared to Choi and Lee (2020) (93.84% accuracy with a DCNN ensemble), Chen et al. (2025) (97.65% accuracy, 96.98% precision, 96.40% recall with MMDF), and Haq et al. (2025) (92.3% accuracy, 92.1% precision with CNN-LSTM), the proposed model achieves substantial improvements of 5.54, 1.73, and 7.08%, respectively, in the Acc metric. These results fully demonstrate the superior capability of the proposed GGDA framework in modeling complex structural features.

For the four-class task, the proposed model achieves 99.28% accuracy, 99.22% precision, 99.11% recall, and 99.17% F1-score, ranking second only to the SCCAN method by Hassan et al. (99.22% accuracy). It is worth noting that the SCCAN method was published in 2025, and the proposed model achieves highly comparable performance with a marginal difference of only 0.06% in accuracy, while showing trade-offs in other metrics. More importantly, the proposed model significantly outperforms other compared methods: compared to Loddo et al. (2022) (97.71% with Deep-Ensemble), Al-Adhaileh et al. (2022) (94% with AlexNet), Papanna Umalakshmi et al. (2024) (95.45% with ADEMNET), Choudhury et al. (2024) (94.5% accuracy, 95% precision with Coupled-GAN), and Yilmaz and Kuş (2026) (96.43% accuracy with GA-optimized model), the proposed model achieves improvements of 1.57, 5.28, 3.83, 4.78, and 2.85%, respectively.

The proposed framework emphasizes three key contributions: (i) geometry-aware feature learning through LDConv and GA attention; (ii) lightweight and efficient architecture, facilitating deployment in clinical settings; and (iii) improved interpretability, with the ability to highlight anatomical regions relevant to AD progression.

## 6 Conclusion

This paper presents a novel deep learning architecture for Alzheimer’s disease classification based on MRI images. The proposed model integrates LDConv with a Geometry-guided deformable mechanism to enhance feature representation and spatial modeling capability. Specifically, LDConv introduces flexible sampling patterns through learnable offsets, enabling both regular and irregular convolution operations. When the kernel size is set to K^2^, the operation degenerates into standard deformable convolution, providing adaptable geometric feature extraction. Furthermore, the proposed GA attention effectively exploits the geometric information encoded in the offsets to guide the network toward more discriminative regions.

Future work will focus on improving the convolutional design by incorporating attention mechanisms to adaptively regulate feature extraction. Meanwhile, LDConv with more appropriate kernel sizes and sampling shapes will be explored. The proposed framework will also be extended to larger multi-center datasets to further evaluate its robustness and generalization capability.

## Data Availability

The original contributions presented in the study are included in the article/supplementary material, further inquiries can be directed to the corresponding authors.
